# Supplementary material for: Academic achievement after a CT examination toward the head in childhood: Follow up of a randomized controlled trial
Source: PLoS One. 2023 Apr 19;18(4):e0284712. doi: 10.1371/journal.pone.0284712 (PMC10115270; doi:10.1371/journal.pone.0284712)
Supplement: Attachment — Submitted filename: Response to Reviewers.docx [file pone.0284712.s001.docx]

PLOS ONE

PONE-D-22-29193

Academic achievement after a CT examination toward the head in childhood: Follow up of a randomized controlled trial.

March 5, 2023

Dear Reviewers,

Thank you for very much for your corrections and comments, we sincerely believe they have enhanced the quality of the article. We have made the following changes according to your input:

|  | *Reviewer #1: This is an interesting study assessing if ionizing radiation dose from a CT examination of the head at the age of 6-16 years affects academic performance and high school eligibility at the end of compulsory school.* |  |
| --- | --- | --- |
|  |  |  |
| 1 | *As this is a follow-up analysis, in the methods section can the authors be explicit about what will be defined as baseline for analysis.* | **Revised** |
|  | The baseline for this follow-up study is theoretically the most recent school grades at study inclusion. This information would only be available for the participants included at approximately 12 years of age, since the school system in Sweden starts with grades in grade 6. However, since the study is a randomized controlled trial the baseline data is not further analyzed (Senn, S., *Seven myths of randomisation in clinical trials.* Statist. Med., 2013. 32(9): p. 1439-1450 and Moher, D., et al., *CONSORT 2010 Explanation and Elaboration: updated guidelines for reporting parallel group randomised trials.* BMJ, 2010. 340(c869)) and we assume that the grades are similarly distributed between the two groups, i.e. no difference in high or low grades between the exposed to ionized radiation and the non-exposed. As is defined in the CONSORT statement (Moher et al., 2010) we have presented the baseline demographic and clinical characteristics in table 1. This has been made more clear in the results section.  *Results, first paragraph.* |  |
| 2 | *Also mention any handling of missing data - if any approach will be used.* | **Revised** |
|  | Concerning the handling of missing data, we could only have analyzed the group “not yet graduated” (N_total_=33) for possible differences, although we know that they as a group must be younger, have participated most recently (shorter follow up time) than the participants included in the present study, but that they were in equal numbers in the allocated treatment groups. However, a possible problem of this could be that shorter follow up times could affect the results, which is a discussion for the generalizability of the study. As mentioned in the article, first inclusion date was used for the “doubles” and the ones who had “graduated before inclusion” to the original study were excluded from this follow up study. We are not able to analyze the “incomplete PIN numbers” due to missing information nor the “loss to follow up” group, since data is not traceable after linkage by Statistics Sweden. Since there was not a choice of participating, we do not have the problem of selection bias. We have added information about this to the results section.  *Results, first paragraph.* |  |
| 3 | *Mention checking of model assumptions.* | **Revised** |
|  | We have added information and references for the statistical analyses in the methods section.  *Methods, Statistical methods, first paragraph.* |  |
| 4 | *Table 1 - update title e.g Descriptives of participants by exposure of ..... status.* | **Revised** |
|  |  |  |
| 5 | *Table 1 includes footnote "intention to treat" - can authors define this in the methods section.* | **Revised** |
|  |  |  |
| 6 | *Table 1 headers state exposed and unexposed - for easy readability do you mean exposed to ionizing radiation - can this be reflected in the header and in methods section.* | **Revised** |
|  |  |  |
| 7 | *Table 2 - include ranges in your results.* | **Revised** |
|  |  |  |
| 8 | *Logistic regression has been stated the main method of analyses - in the methods state that unexposed is your reference and what the and OR<1 or OR>1 would mean in terms of probability of ?reduced academic performance and high school eligibility.* | **Revised** |
|  | Information has been added according to suggestion.  *Results, second paragraph.* |  |
| 9 | *The results are not surprising, if you observed no differences in baseline characteristics in terms of you variables of interest, both the Crude OR and Multivariate OR. Considering the data is coming from an RCT where in general students were generally comparable only differences based on randomisation group. This should be expanded further in discussion. Why did the authors not consider also adjusting for randomisation group.*  We agree with the reviewer that both the rationale for our decisions about analysis and our findings can be further elaborated in the text. In the original Octopus study, 96.5% received the original treatment (observation v.s. exposure to computed tomography). In addition, 8.9% of the patients randomized to computed tomography were admitted to inhospital observation, and 8.6% of patients received a CT as well. Since these departures from original protocol are highly likely to differ from the rest of the study group in factors such as trauma severity, disease progression and social factors that may be related to outcome, we think that we may introduce a systematic error, and hence loose the advantages given by the randomization of radiation exposure, if we do not analyze the data according to intention to treat. Given the strong correlation between randomization group and exposure we abstained from introducing both these variables in the model. Information about this has been added to the discussion.  *Discussion, third paragraph.* | **Revised** |
|  |  |  |
|  | *Reviewer #2: Thank you for the opportunity to review this manuscript! This is a fantastic study to address concerns of low-level radiation. There are some issues I would like to discuss and have address prior to publication.* |  |
|  |  |  |
| 1. | *Overall, I think the article fails to address and control for care of the subjects following their mild traumatic brain injury (mTBI). If a patient was discharged with a mTBI, what is the likely hood they followed the physician's instructions compared to the patients that were held for observation. A likely cause of the difference between exposed and non-exposed subjects was that the discharged patients did not properly follow their treatment plan resulting in improper healing. The study should include some way of controlling for the difference in the standard of care. One possible way of doing this would be comparing the results to subjects who did not experience a mTBI to see if the change in scores was consistent between exposed and non-exposed subjects.* | **Revised** |
|  | We are agree with the reviewer that this may a problem. Given the design of the original OCTOPUS-study, however, it is impossible to disentangle the effects from hospital stay with the effects from CT exposure (The Evidence Base of Clinical Diagnosis: Theory and Methods of Diagnostic Research, 2nd Edition  [J. André Knottnerus (Editor)](https://www.wiley.com/en-us/search?pq=%7Crelevance%7Cauthor%3AJ.+Andr%C3%A9+Knottnerus), [Frank Buntinx (Editor)](https://www.wiley.com/en-us/search?pq=%7Crelevance%7Cauthor%3AFrank+Buntinx)  ISBN: 978-1-405-15787-2 November 2008 BMJ Books). To remedy this, however, we address the question of whether children with mild head trauma differ from other children by comparing all the study subjects with their, supposedly, unexposed siblings. This, however, is a separate study outside of the scope of the present one, but we have added information out methodological constraints to the discussion.  *Discussion, second to last paragraph* |  |
| 2. | *In the “Radiation dose reconstruction” section, second to last paragraph, it is mentioned that the lowest reconstructed CTDI_vol was used. It is far more common to use the highest reconstructed dose. Using the highest dose represents the worst-case scenario that a patient could receive which is generally the main concern in the health physics field.* | **Revised** |
|  | In general the reconstructed CTDI_(vol, head)_ represents the average dose, but yes, the lowest CTDI_(vol, head)_ was used in cases where there was uncertainty about the approximated collimation width. We chose to do like this since any misclassification of dose where patients exposed to lower doses were to be allocated to higher exposure groups would risk leading to an underestimation of any negative effects. This is now explained more clearly in the text.  *Methods, Radiation dose reconstruction, third paragraph* |  |
| 3. | *Additionally, in the last paragraph mentions erroneous metadata. Some more information here on what criteria was used to define erroneous metadata would be beneficial.* | **Revised** |
|  | We tried to get all available information from the sources available to get as many doses reconstructed with the same method. With erroneous metadata we mean that the data could not be extracted from any of the sources, not that we excluded any data available. What constitutes erroneous or lacking metadata has been made more clear in the text.  *Methods, Radiation dose reconstruction, last paragraph* |  |
|  | *Reviewer #3: For Materials and methods section, more reasoning should be included.* |  |
|  |  |  |
| 1. | *Why is the study done in such small size population?* | **No revision** |
|  | Our knowledge of the effects on cognition from ionizing radiation generally comes from much smaller studies than the present, and because of ethical reasons, randomized studies on radiation exposure are naturally rare. Therefore, the population size, although being small compared to traditional register studies, is exceptional for a randomized study in the field of radiation exposure. The advantages and limitations of the study are presented in the discussion section, the biggest shortcoming due to small population size being limitations in finding statistically significant results for possible small differences, and it is also why our conclusion of the study is not that there is no effect on cognition from low dose radiation, but if there is, it is too small to be detected “…in a study of more than 800 study subjects, half of whom were randomly assigned to computed tomography. |  |
| 2. | *What are the shortcomings of methodology of the study?* | **Revised** |
|  | Please see answer to Reviewer 2, question 1. |  |
| 3. | *What are the reasons behind applying the methods for this study? Questions like these need to be answered in the Materials and methods section. Also, for the statistical methods why Chi-Square Test and Student’s t-Test were performed to analyze the data must be explained in detail.* | **Revised** |
|  | The statistical methods used in the study are common within epidemiological research and describing them in detail are not within the scope of the present study. However, as mentioned above (Reviewer 1, questions 1-3), we have added information and references of the statistical methods used. |  |
| 4. | *For the results section I think some calculations of the statistical analysis should be included.* | **No revision.** |
|  | Please see answer 3 above. |  |
|  |  |  |
|  |  |  |
| 5. | *Some graphical representation of the results might be better for understanding the results of the study.* |  |
|  | We have chosen to present the results in a similar way across the different analyses to keep the presentation coherent. Given the number of outcome variables and that we found no association between these outcome variables and exposure, we found for the whole that table-presentation was both the clearest and the most space efficient way to present our data.  *Journal requirements*  The new references in the article have been added to the reference list, the references in question are numbered 20-24. In addition, we have updated “Figure 1” to meet PLOS ONE’s style requirements. Furthermore, we have included the ethics statement to the first part of the Methods section. | **No revision.** |

We hope that the changes and the revised manuscript will be to your satisfaction and appropriate for publication in PLOS ONE.

Kind Regards,

Elina Salonen
